# An unusual camellia-shaped lesion in a female with diarrhea: beauty is only skin deep

**DOI:** 10.1055/a-2584-1349

**Published:** 2025-05-22

**Authors:** Yi Lu, Jiachen Sun, Weihao Deng, Jiancong Hu

**Affiliations:** 1 373651 Department of Gastrointestinal Endoscopy, The Sixth Affiliated Hospital, Sun Yat-sen University, Guangzhou, China; 2 Guangdong Provincial Key Laboratory of Colorectal and Pelvic Floor Diseases, The Sixth Affiliated Hospital, Sun Yat-sen University, Guangzhou, China; 3 Biomedical Innovation Center, The Sixth Affiliated Hospital, Sun Yat-sen University,, Guangzhou, China; 4 Department of Pathology, The Sixth Affiliated Hospital, Sun Yat-sen University, Guangzhou, China

A 40-year-old woman was admitted due to chronic diarrhea for 6 months. Her laboratory tests revealed anemia (hemoglobin 104 g/L) and positive for fecal occult blood test. Enhanced chest and abdominal computed tomography scans demonstrated multiple enlarged lymph nodes at the root of the mesentery ( Fig. 1 , yellow arrows), with the largest measuring 17 mm in short-axis, splenomegaly, and pulmonary nodules. Gastroscopy showed nonatrophic chronic gastritis and fundic gland polyps. Colonoscopy identified multiple camellia-shaped lesions in the terminal ileum ( Fig. 2 **a** ), and magnified endoscopy (ME) with narrow-band imaging (NBI) showed opaque micro-elevation with superficial, thin branch-like vessels ( Fig. 2 **b** ). Multiple small, flat lesions were diffusely distributed throughout the entire colon ( Fig. 2 **c** ), resembling nodular lymphoid hyperplasia 1 , but with a distinctive feature: the lesions exhibited not only a reddish outline but also a reddish central area ( Fig. 2 **d** ). In the lower rectum, multiple slightly elevated lesions were observed ( Fig. 2 **e** ), and ME-NBI again revealed opaque micro-elevations with superficial, thin branch-like vessels ( Fig. 2 **f** , Video 1 ). The biopsy pathology showed nodular lymphoid hyperplasia in the intestinal mucosa, with nuclei appearing round or irregular ( Fig. 3 **a, b** ). Immunohistochemistry results showed positive for CD20 ( Fig. 3 **c** ), CD21 (indicating follicular dendritic cells, Fig. 3 **d** ), CD10 ( Fig. 3 **e** ), and Bcl-6 ( Fig. 3 **f** ), while CD3 and Bcl-2 were negative, and Ki-67 was positive in 30% of cells; CD43 showed partial positive. Further B-cell clonality assessment detected a monoclonal rearrangement. The patient was ultimately diagnosed with follicular lymphoma (grade 3A, Ann Arbor stage IVA, FLIPI-2 score 3, and high risk), and underwent chemotherapy. This case highlights the striking endoscopic appearance of intestinal follicular lymphoma, underscoring the importance of recognizing such lesions, despite their potentially deceptive benign appearance.

Endoscopy_UCTN_Code_CCL_1AD_2AC

**Fig. 1:**
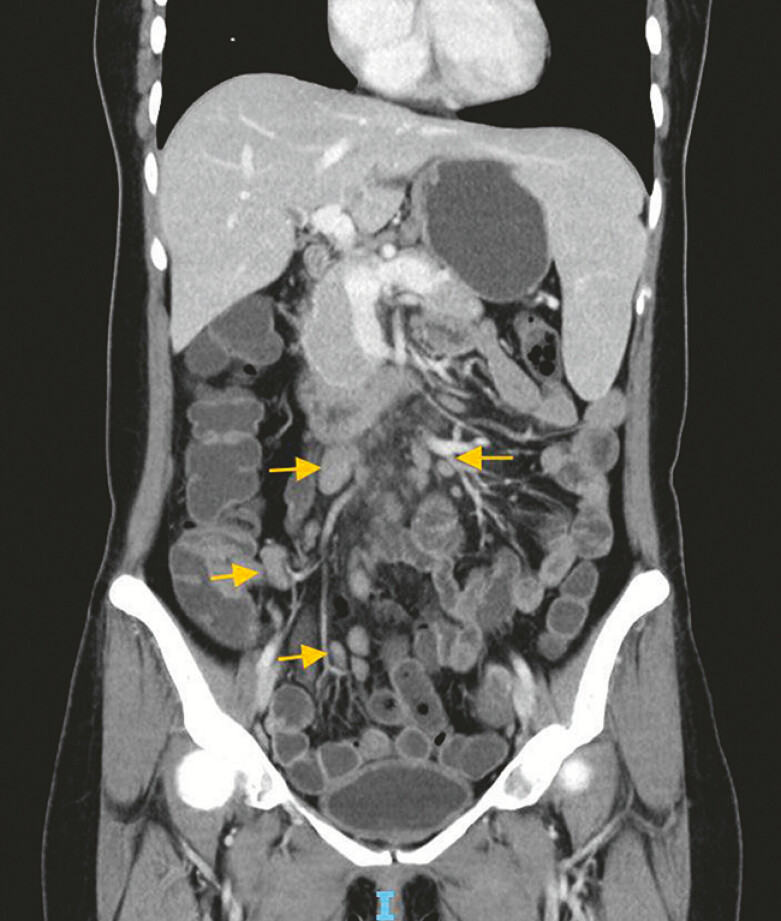
Enhanced computed tomography scans demonstrated multiple enlarged lymph nodes at the root of the mesentery.

**Fig. 2:**
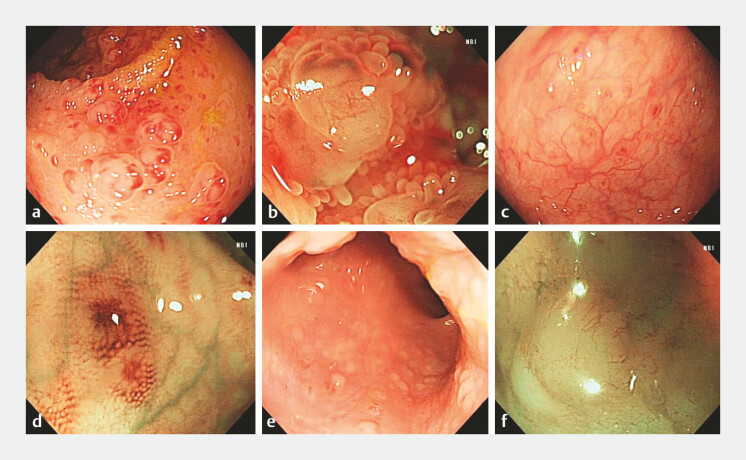
Colonoscopy images of the lesions: **a** multiple camellia-shaped lesions in the terminal ileum; **b** ME-NBI showed opaque micro-elevation with superficial, thin branch-like vessels; **c** the entire colon was diffusely distributed with multiple small, flat lesions; **d** the lesions resembling nodular lymphoid hyperplasia, but with a distinctive feature: the lesions exhibited not only a reddish outline but also a reddish central area; **e** multiple slightly elevated lesions were observed in the lower rectum; **f** ME-NBI revealed opaque micro-elevations with superficial, thin branch-like vessels. Abbreviation: ME-NBI, magnified endoscopy with narrow-band imaging.

**Fig. 3:**
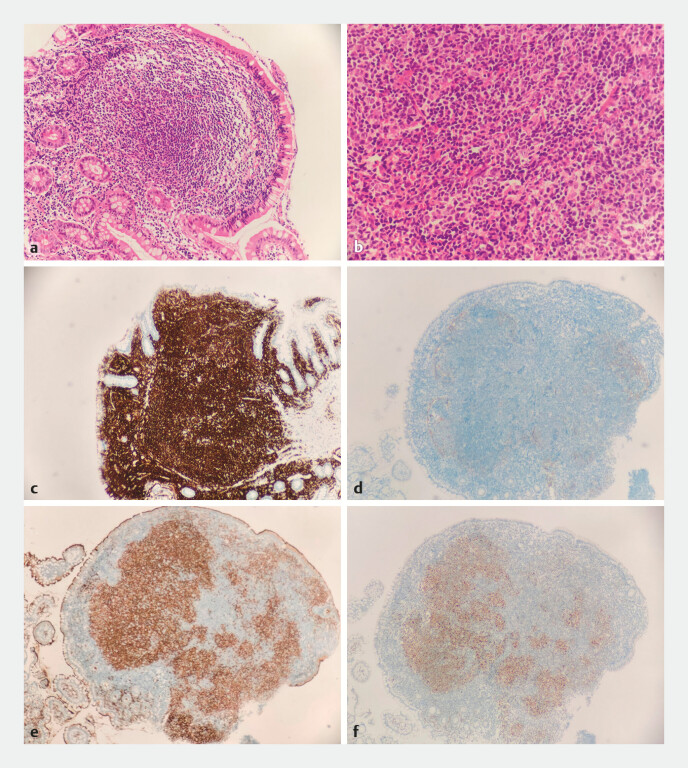
Pathology images of the lesions (terminal ileum): **a, b** nodular lymphoid hyperplasia in the intestinal mucosa, with nuclei appearing round or irregular; **c** immunohistochemistry results CD20 (+); **d** CD21 (+) in follicular dendritic cells; **e** CD10 (+), **f** Bcl-6 (+).

Colonoscopy images of an unusual camellia-shaped lesion in a woman with diarrhea.Video 1

## Notes

Endoscopy E-Videos https://eref.thieme.de/e-videos
*E-Videos* is an open access online section of the journal *Endoscopy* , reporting on interesting cases and new techniques in gastroenterological endoscopy. All papers include a high-quality video and are published with a Creative Commons CC-BY license. Endoscopy E-Videos qualify for HINARI discounts and waivers and eligibility is automatically checked during the submission process. We grant 100% waivers to articles whose corresponding authors are based in Group A countries and 50% waivers to those who are based in Group B countries as classified by Research4Life (see: https://www.research4life.org/access/eligibility/ ). This section has its own submission website at https://mc.manuscriptcentral.com/e-videos .
